# David James Jolley BSc, MBBS, DPM, MSc, FRCPsych

**DOI:** 10.1192/bjb.2024.73

**Published:** 2025-02

**Authors:** Claire Hilton

Formerly Consultant Psychiatrist and Honorary Reader at the Personal Social Services Research Unit, Manchester, UK; and Consultant Psychiatrist, Medical Director and Honorary Professor of Psychiatry, Wolverhampton, UK

**Figure:**
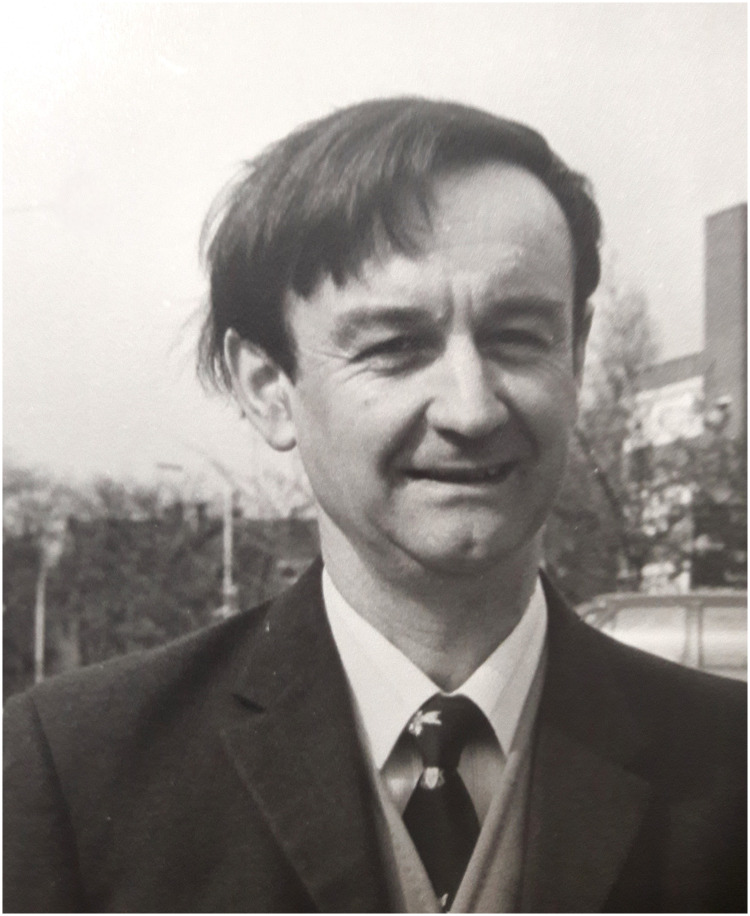

Dave Jolley, who died aged 79 on 17 May 2024, established the pioneering South Manchester Old Age Psychiatry Service in 1975 and led it for the next 20 years. He was then Medical Director and Honorary Professor of Psychiatry in Wolverhampton. Following that, he turned to devising other ground-breaking older people's mental health community services in Manchester and Staffordshire, repeatedly demonstrating what could be done to improve people's lives.

Dave also took leading roles on policy and practice committees across the Royal College of Psychiatrists, the Royal College of Physicians and the British Geriatrics Society. He was one of a small band who campaigned for Department of Health recognition of ‘psychogeriatrics’ as a distinct medical specialty (achieved 1989). His research and publications spanned numerous aspects of old age psychiatry, including epidemiology, spirituality, ethnicity, care homes, palliative care and suicide.

Born on 9 December 1944, Dave grew up in Woodland Crescent, Wolverhampton, a street of modest 1930s semi-detached houses, with many of his closeknit, working-class family nearby. They had moved to the new suburbs from one of the poorest areas of industrial Wolverhampton, known as Hell Lane, Ettingshall, near the furnaces and open mining. Even after moving away, Ettingshall's George Street Methodist Church remained a focus of family life. His aunt, Annie, taught at the Sunday school, and Dave and his younger brother Malcolm attended. Dave remained inspired lifelong by his Christian faith and Methodist ideals.

Dave's parents – James and Hilda (née Cooke) – had little formal education. James worked for British Oxygen, as a delivery driver and storeman, and Hilda was a housewife. They sought better for their sons. Dave got a place at Wolverhampton Grammar, a boys’ state school. Around that time, he also had his first taste of psychiatry. Grandma Jolley had dementia and he visited her each week in Stafford mental hospital. He recalled in 2023: ‘you'd have to be let in with keys … and you'd sit in this big room, at a table, and she'd talk absolute nonsense about times way before she'd been married’.

Dave studied medicine at Guy's Hospital, London, and decided to be a psychiatrist early on. His student elective with Dr Russell Barton, who had transformed Severalls psychiatric hospital from largely custodial to a progressive, community-focused institution, inspired him as to what could be achieved. Shortly before graduating in 1969, Dave married Janis O'Connor, a nurse. They had three children, Ben, Kate and Esther, but the marriage ended in divorce in the early 1980s.

After house jobs in Jersey and the Isle of Wight, Dave returned to the mainland to pursue his career in psychiatry. He arrived at Withington Hospital, Manchester, shortly before the opening of its prestigious new Psychiatric Unit, symbolizing the future of academic and community psychiatry away from the large mental hospitals. Intent on being an old age psychiatrist when the specialty was a mere vision in the eyes of a few enthusiasts and no formal training scheme existed, he undertook secondments with old age psychiatrists Felix Post at the Maudsley and Tom Arie at Goodmayes, and with geriatrician John Brocklehurst in Manchester, and visited innovative services in Southampton and Newcastle upon Tyne.

In 1975, aged 30, Dave was appointed first consultant old age psychiatrist in the NHS North Western region. A Victorian workhouse pavilion became Withington's Psychogeriatric Unit. In addition, each of the five general psychiatrists gave him two beds in their new building, although only two were vacant: the others came with their patients, ‘old people that they'd got stuck with,’ Dave said. The Manchester psychiatric establishment – among them, Professors David Goldberg and Neil Kessel – looked on in awe as his comprehensive community, day hospital and inpatient services flourished. Enthusiastic colleagues of different disciplines sought to join his team. Outpatient clinics meant all the doctors going to visit patients at home, returning to a meeting to discuss them. That usually took place in Dave's office where a wall was adorned with Wolverhampton Wanderers posters. By the mid-1990s, Dave had trained almost every consultant old age psychiatrist in the region and many further afield.

While Dave was chair of the RCPsych Section (now Faculty) of Old Age Psychiatry (1989–1994), the number of ‘consultant psychogeriatricians’ in the UK grew from around 280 to over 400. As chair, he learnt that Wolverhampton was one of four districts in the country with no old age service, so he went there to set one up. He was delighted to return to his family roots and many good memories. Just as when he started at Withington, he was given carte blanche to develop services, and as previously, his schemes flourished.

A crunch came in 2002 when Dave required three cardiac stents. He thought he should take a quieter lifepath, so continued just with clinical work in Wolverhampton for another two years. He then returned to work in Manchester, clinically and at the University Personal Social Services Research Unit. Then, at Tameside General Hospital and Willow Wood Hospice, he facilitated a new joint palliative care and mental health service for people with dementia, and in Gnosall, Staffordshire, he collaborated with general practitioners to establish a primary care memory clinic. Right until the end of his life, he worked tirelessly with voluntary organisations, giving them the benefit of his vast experience.

With dedication, determination, humility and humanity, Dave inspired many people professionally and in the community in Altrincham where he lived with his second wife Susan (née Hodgson, a psychiatrist; married 1985), and where they brought up their daughters Emily and Sarah. He contributed tirelessly to his local Methodist Church and to making John Leigh Park an award-winning local amenity. He was made a Freeman of the Borough; his certificate was inscribed: ‘To the worthy Professor Dr David Jolley, Gentleman of the Barony of Dunham Massey’.

When asked what he would say to someone stepping into his very large footprint, Dave initially replied that he took size five shoes, so his footprint was only small. Then he said:

… live a life which is good, respectful, driven by love of others and supported by love of God. Not everyone will take the latter, but it is there in me. Go into every situation with an open mind, preparedness to learn, to gain understanding, to work with others of like-mind and to take action which will make a difference for the better – sometimes against all odds, often against all expectations.

Dave practised what he preached.

He died peacefully at home in the care of his family.

